# Clinical significance of the tumor microenvironment on immune tolerance in gastric cancer

**DOI:** 10.3389/fimmu.2025.1532605

**Published:** 2025-02-14

**Authors:** Xiangyang He, Xin-Yuan Guan, Yan Li

**Affiliations:** ^1^ State Key Laboratory of Oncology in South China, Guangdong Provincial Clinical Research Center for Cancer, Sun Yat-sen University Cancer Center, Guangzhou, China; ^2^ Department of Clinical Oncology, The University of Hongkong, Hong Kong, Hong Kong SAR, China

**Keywords:** gastric cancer, immunosuppression, tumor microenvironment, immunotherapy, metabolize

## Abstract

In the realm of oncology, the tumor microenvironment (TME)—comprising extracellular matrix components, immune cells, fibroblasts, and endothelial cells—plays a pivotal role in tumorigenesis, progression, and response to therapeutic interventions. Initially, the TME exhibits tumor-suppressive properties that can inhibit malignant transformation. However, as the tumor progresses, various factors induce immune tolerance, resulting in TME behaving in a state that promotes tumor growth and metastasis in later stages. This state of immunosuppression is crucial as it enables TME to change from a role of killing tumor cells to a role of promoting tumor progression. Gastric cancer is a common malignant tumor of the gastrointestinal tract with an alarmingly high mortality rate. While chemotherapy has historically been the cornerstone of treatment, its efficacy in prolonging survival remains limited. The emergence of immunotherapy has opened new therapeutic pathways, yet the challenge of immune tolerance driven by the gastric cancer microenvironment complicates these efforts. This review aims to elucidate the intricate role of the TME in mediating immune tolerance in gastric cancer and to spotlight innovative strategies and clinical trials designed to enhance the efficacy of immunotherapeutic approaches. By providing a comprehensive theoretical framework, this review seeks to advance the understanding and application of immunotherapy in the treatment of gastric cancer, ultimately contributing to improved patient outcomes.

## 1 Introduction

Gastric cancer (GC) continues to be one of the most prevalent and clinically significant gastrointestinal malignancies globally, characterized by elevated morbidity and mortality rates (1). For early-stage gastric cancer, surgical resection is the primary treatment modality (2). However, in advanced-stage disease, surgical intervention is often not feasible. In such cases, the standard of care involves chemotherapy regimens, typically combining fluoropyrimidine and platinum-based agents, or second-line therapies such as paclitaxel and irinotecan. Unfortunately, these treatments have shown limited efficacy in improving overall survival (3, 4). The advent of immunotherapy has introduced a promising new avenue for the treatment of gastric cancer. Trastuzumab, a monoclonal antibody targeting human epidermal growth factor receptor 2 (HER2), has demonstrated a significant improvement in overall survival among HER2-positive gastric cancer patients (5). However, the therapeutic applicability of HER2-targeted therapy is limited, as only 15-30% of gastric cancer patients express HER2 positivity, leaving a substantial portion of patients without effective treatment options (6, 7). Recent studies have also explored the use of programmed death ligand 1 (PD-L1) monoclonal antibodies, which have shown superior outcomes compared to first-line chemotherapy (8). Nevertheless, the therapeutic benefits remain modest, potentially due to the challenges posed by immune tolerance within the tumor microenvironment.

The tumor environmental (TME) includes non-tumor components and their metabolic or secretory products, for example, a diverse population of immune cells and stroma cells. In the early stages of tumor development, the infiltration of immune cells plays a crucial role in mounting an effective anti-tumor response, significantly impeding tumor progression (9). However, as the tumor progresses, the initially tumor-suppressive microenvironment manifests itself in an immunosuppressive state that impairs the anti-cancer immune response and fosters immune tolerance (9, 10). The stomach has a strong acidic environment, a unique endocrine system and a microbiota, which makes the TME of GC different compared to other solid tumors. For instance, altered acid secretion disrupts the balance of the gastric microbiome, and it has been found that the microbiota of patients with GC has fewer microorganisms responsible for the production of short-chain fatty acids, resulting in a decrease in butyrate (11, 12). Butyrate, however, has the ability to enhance CD8^+^ T cell function by acting as a homology box with the G protein-coupled receptor 109A/homology domain protein (12). Therapeutic strategies targeting the TME aim to counteract the immunosuppressive state and remodel the TME, thereby reactivating the patient’s immune response to sustain its anti-tumor activity.

In this review, we outline the mechanisms by which the GC TME induces immune tolerance and discuss recent advances in understanding how the components of the TME can be reprogrammed to stimulate an immune response. Summarizing and analyzing the potential mechanisms of GC immunosuppressive microenvironment formation can provide a theoretical basis and new therapeutic approaches to improve the clinical treatment of gastric cancer. Specifically, we summarize current therapies for GC immunosuppression, aiming to provide improved directions for further drug development to reverse the immunosuppressive microenvironment and identify new targets.

## 2 Components of the tumor microenvironment

### 2.1 Tumor-associated macrophages

Macrophages that infiltrate the tumor microenvironment are referred to as tumor-associated macrophages (TAMs) and generally exist in two distinct phenotypes. One is the classically activated M1-like macrophage, which promotes an anti-tumor immune response through the production of pro-inflammatory cytokines such as IL-12, IL-1, IL-6, and tumor necrosis factor (TNF)-α, thereby exerting tumor-suppressive effects (13, 14). The other is the alternatively activated M2-like macrophage, induced by IL-13 and IL-4, which plays a role in supporting tumor growth, tissue remodeling, and promoting tumor progression (15, 16). Recent studies have delved into the molecular mechanisms underlying M2-like macrophage activation in gastric cancer. Cui’s study reveals that Pentraxin-3 (PTX3) effectively inhibits the stemness of GC cells and modulates the TME by preventing the M2-polarization of macrophages which is known to promote tumor progression and immune evasion (17). Furthermore, the high mobility group A 1B/2 was shown to upregulate the expression of POU class 1 homeobox 1 (POU1F1), which regulates M2-like macrophage polarization via the CXCL12/CXCR4 signaling axis, and contributes to the metastasis of gastric cancer to the lungs (18).

Recently, TAMs have emerged as a promising therapeutic target in cancer treatment. Current therapeutic strategies targeting TAMs include several approaches: inhibiting TAMs recruitment to tumor sites, depleting TAMs population, reprogramming TAMs to adopt a pro-inflammatory M1 phenotype, and enhancing their phagocytic activity (19). Each strategy is designed to mitigate the immunosuppressive and tumor-promoting functions of TAMs, thereby restoring their antitumor potential and improving therapeutic efficacy. A phase 1 trial in patients with HER2- overexpressing solid tumors, including GC, have achieved therapeutic efficacy with adenoviral-transduced autologous macrophages containing anti-HER2 chimeric antigen receptors (20). In an open-label, single-arm, phase 2 trial, patients with advanced GC were given 20 mg of lenvatinib (significantly reduces TAMs and increases CD8^+^ T-cell infiltration) orally daily and 200 mg of pembrolizumab intravenously every 3 weeks until disease progression, intolerable toxicity, or withdrawal of consent (21). Lenvatinib in combination with pembrolizumab demonstrated promising anti-tumor activity and an acceptable safety profile (21). Another trial indicated that Bexmarilimab (1.0 mg/kg every 3 weeks) induced macrophage activation resulting in better therapeutic efficacy and was well tolerated in GC patients (22). Sitravatinib is a tyrosine kinase inhibitor targeting TAM, and a multi-cohort phase 1b/2 clinical study demonstrated that sitravatinib (120 mg orally once daily) resulted in a modest improvement in objective remission rates of GC and was generally well tolerated (23). In addition, a Phase 1 clinical trial evaluating the efficacy of targeting Claudin 18.2 and CD47 in patients with advanced gastric and gastroesophageal junction adenocarcinoma is undergoing (24). Collectively, these clinical trials provide promising approaches for targeting TAMS and modulating TME of GC. With a deeper understanding of the biology of TAMs and their interactions with the TME, innovative strategies to harness or modulate TAMs will crucial in advancing GC therapy.

### 2.2 T-cells

Within the tumor microenvironment, T cells exhibit significant heterogeneity, playing diverse roles in the immune response to cancer. CD8^+^ T cells are a key component of the anti-tumor immune response, recognizing tumor antigens presented by MHC class I molecules on cancer cells. These cytotoxic T cells exert their tumor-killing effects through multiple mechanisms, including the release of granule-associated cytokines, induction of necrosis or apoptosis via ligand-receptor interactions, and secretion of pro-inflammatory cytokines such as interferon-γ (IFN-γ) and TNF-α, which mediate direct cytotoxicity against tumor cells (25, 26). The function of CD8^+^ T cells is typically impaired in GC patients. For instance, toll-like receptor 2 is downregulated in CD8^+^ T cells from GC patients, which affects the expression of perforin and granzyme B, leading to decreased cytotoxicity (27).

Regulatory T cells (Tregs) play a critical immunosuppressive role within the TME, dampening the anti-tumor immune response and facilitating immune evasion of tumor cells. Tregs produce a variety of immunosuppressive cytokines, including transforming growth factor-beta (TGF-β), which suppresses the cytotoxic function of CD8^+^ T cells and Tregs indirectly promotes the polarization of macrophages towards the tumor-supportive M2 phenotype (28–30). In addition, TGF-β also exerts a promoting effect on Treg cell production (31). Moreover, Tregs inhibit the expression of MHC class II molecules on dendritic cells (DCs) via the expression of lymphocyte activation gene-3 (LAG-3) (32, 33). In an *in vitro* model, Treg cells are enriched in early intestinal GC and can induce interleukin-2Rα expression and activation of the mitogen-activated protein kinase (MAPK) signaling pathway in tumor cells, leading to the growth of tumor cell spheroids (34). The level of infiltration of tumor necrosis factor receptor 2-positive Tregs increases with the progression of GC, and the expression of the immunosuppressive phenotype and function of Tregs is closely related to the activation of the TNF-α/TNFR2 pathway (35).

Therapies aimed at enhancing CD8^+^ T cells activity and reducing Treg-mediated suppression could significantly improve immune-mediated tumor control, leading to more effective and durable treatment responses in cancer patients. For instance, the application of TGF-β inhibitors can release the inhibitory function of TGF-β on CD8^+^ T cells, and reduce the production of Treg cells (28, 29, 31). As we continue to advance our understanding of the molecular mechanisms underlying the CD8^+^ T cell-Treg axis, novel therapeutic interventions are likely to emerge, offering new hope for the treatment of cancers that have been resistant to immunotherapy.

### 2.3 Natural killer cell

Tumor cells employ multiple strategies to evade immune surveillance, particularly the cytotoxic activity of CD8^+^ T cells during tumorigenesis and progression. However, natural killer (NK) cells provide a complementary arm of the immune response, as they can directly target and eliminate tumor cells without the need for prior antigen presentation (36). Despite this critical role, the function of NK cells is notably impaired in patients with GC. Studies have shown a significant increase in NK cell apoptosis in these patients, and the extent of NK cell apoptosis correlates with the progression and severity of the disease (37). Natural killer Group 2 Member D (NKG2D) is a key receptor for the activation of NK cells, and it has been found that the expression of NKG2D in patients with gastric cancer has been positively associated with improved clinical outcomes, including better overall survival (OS) (38). In addition, GC leads to elevated serum IL-10 and TGF-β1 levels, which have an inhibitory effect on NK cytotoxicity (39). Recently, it has also been shown that prostaglandin E2 (PGE2) produced by GC cells inhibits the proliferation of NK cells (40). NK cell-based therapies demonstrated substantial efficacy in the treatment of hematological malignancies and some solid tumors (41, 42). However, clinical trials specifically evaluating NK cell-based therapies in GC remain absent. Beyond direct NK cell relay therapies, research has indicated that enhancing NK cell-mediated mechanisms may offer therapeutic benefit for GC patients. For example, a trial investigating trastuzumab (Herceptin), a humanized monoclonal antibody targeting HER2/neu, demonstrated its capacity to improve antibody-dependent cellular cytotoxicity mediated by NK cells, suggesting a synergistic role in immunotherapy (43). Additionally, bemarituzumab is a humanized IgG1 monoclonal antibody specific to the splice-variant FGFR2b. It is also glycoengineered for increased affinity for the human Fc-γ RIIIA receptor expressed on NK cells, enabling enhanced antibody-dependent cell mediated cytotoxicity (44). A phase I study of bemarituzumab in FGFR2b-overexpressing GC patients revealed promising outcomes, with a favorable safety profile and notable antitumor activity (44). Given the vital role of NK cells in anti-tumor immunity, therapeutic strategies aimed at restoring or enhancing NK cell function, either by boosting NKG2D activity or by counteracting the immunosuppressive effects of cytokines and PGE2, could also offer promising new avenues for the treatment of gastric cancer.

### 2.4 Neutrophils

Neutrophils, traditionally recognized for their role in host defense, possess the ability to inhibit tumor growth through various mechanisms. These include the release of antimicrobial and cytotoxic compounds that can directly eliminate malignant cells, as well as the secretion of cytokines and chemokines that recruit additional immune cells with anti-tumor activity (45). However, neutrophils may also play a paradoxical role in promoting tumor progression. In untreated tumor-bearing models, neutrophils have been observed to exert pro-tumorigenic effects, classifying them as N2-type neutrophils. This pro-tumor polarization, however, can be reversed through the blockade of TGF-β, a pivotal modulator in neutrophil polarization. Upon TGF-β depletion, neutrophils adopt an N1 phenotype, associated with robust anti-tumor functions (46). TGF-β thus emerges as a crucial factor in the polarization of neutrophils within the TME. The tumor-suppressive activities of N1-type neutrophils involve various mechanisms, including direct cytotoxicity against tumor cells, inhibition of metastatic spread, induction of tumor cell apoptosis, and reinforcement of anti-tumor immune responses (47). Conversely, N2-type neutrophils facilitate tumor progression via various pathways: remodeling the extracellular matrix to enable tumor invasion, promoting angiogenesis to support tumor growth, and producing neutrophil extracellular traps (NETs) that bolster tumor cell survival and metastatic potential. N2 neutrophils also interact with other immune cells within the TME, fostering immune tolerance and dampening anti-tumor immunity (48). This phenotypic transition has significant clinical implications, particularly in GC, where increased neutrophil infiltration is associated with a higher risk of lymph node metastasis and poorer prognosis (49). Given the dual nature of neutrophil activity in cancer, there is a critical need for the development of safe and targeted therapeutic strategies aimed at selectively inhibiting the tumor-promoting functions of N2-type neutrophils. Such approaches must carefully avoid the potential risks of neutropenia, which could lead to increased susceptibility to infections and exacerbate disease severity. Innovative treatments that can precisely modulate neutrophil function may offer promising avenues for enhancing immune-mediated control of gastric cancer.

### 2.5 Cancer associated fibroblasts

Cancer-associated fibroblasts (CAFs) play a pivotal role in modulating the TME through their dual capacity to synthesize and degrade extracellular matrix (ECM). Additionally, they secrete a wide array of cytokines, chemokines, and exosomes, significantly influencing immune dynamics within the TME. In the context of gastric cancer, patient subgroup with high CAFs score exhibits markedly elevated proportions of monocytes, M2 macrophages, and resting mast cells (50). Single-cell RNA sequencing revealed CAFs associated with poor prognosis of GC patients, with inflammatory CAFs interacting with T cells and extracellular matrix CAFs associated with M2 macrophages (51). Recent experimental evidence has corroborated the interplay between CAFs and macrophages, as well as their impact on the efficacy of immunotherapeutic strategies (52, 53), suggesting that CAFs is involved in the construction of an immunosuppressive microenvironment favorable to tumor development. In addition, CAFs impair the cytotoxic function of NK cells in GC by inducing ferroptosis (54). Therapeutic regimens targeting CAFs have been validated in a variety of tumors (55). However, how to precisely target CAFs for GC treatment are still lacking.

### 2.6 Other components

**Figure 1:**
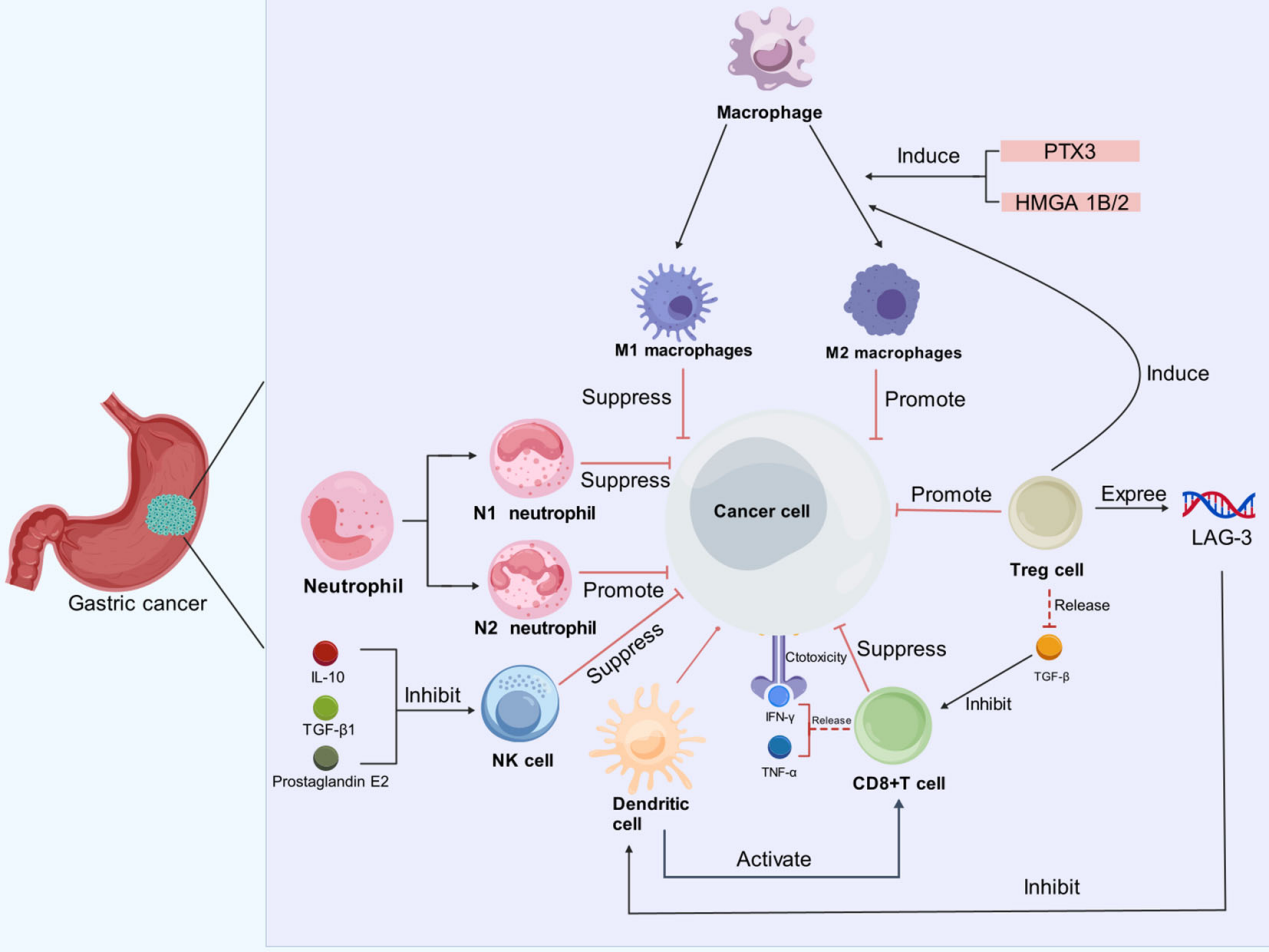
Immune cells exert diverse effects on GC cells in the TME. In the TME of GC, immune cells such as neutrophils, macrophages, dendritic cells, Tregs and CD8^+^ T cells, interact with cancer cells in complex ways. Neutrophils can be polarized into N1 (tumor-suppressive) and N2 (tumor-promoting) phenotypes; Macrophages can differentiate into M1 (anti-tumor) and M2 (pro-tumor) subtypes, influenced by factors like PTX3 and HMGA1B/2. M2 macrophages and Tregs facilitate cancer progression by dampening immune responses. Additionally, prostaglandin E2, IL-10, and TGF-β inhibit the cytotoxic activity of NK cells. Although CD8^+^ T cells produce IFN-γ and TNF-α for their cytotoxic functions, their activity is inhibited by TGF-β. Dendritic cells play a role in activating CD8^+^ T cells, but their function is compromised by the presence of LAG-3.

In addition to above-mentioned cell populations, the TME encompasses critical non-immune components, including ECM and endothelial cells. Significant alterations in the ECM are frequently observed in GC. The ECM becomes progressively more deposited, with increased density, which fosters tumor proliferation, invasion, and metastasis (56). These changes in the ECM not only provide structural support for tumor cells but also create a pro-tumorigenic environment that enhances the aggressiveness of the malignancy. Endothelial cells also play a pivotal role in the TME, particularly through their involvement in angiogenesis, which supplies nutrients to the growing tumor and facilitates its expansion (57). Beyond their role in vascularization, tumor endothelial cells contribute to immune tolerance by inhibiting T-cell activation, reducing the number of cytotoxic CD8^+^ T cells, and promoting the expansion of Tregs (58, 59). The specific contributions of the ECM and endothelial cells in gastric cancer have not been fully elucidated, that underscores the need for further experimental investigation to clarify their roles and to explore their potential as therapeutic targets. Comprehensive studies focusing on the interaction of the stromal elements with the immune system and tumor cells in GC will be essential to develop more effective treatments aimed at remodeling the TME for therapeutic benefit (**Figure 1**).

## 3 Mechanisms of immune tolerance in GC

### 3.1 Glycolysis in TME

Tumor cells predominantly rely on glycolysis for energy production, in contrast to normal non-tumor cells, which utilize oxidative phosphorylation (60, 61). This enhanced glycolytic activity in cancer cells results in inefficient glucose utilization within the TME, a characteristic closely associated with malignant behaviors, including the progression of GC (62). Specifically, the overexpression of Yes-associated protein 1 (YAP1) in GC has been shown to upregulate glucose transporter protein 3 (GLUT3) in macrophages, leading to an accelerated glycolytic metabolism (63). Concurrently, YAP1-induced secretion of IL-13 promotes the polarization of macrophages towards the M2 phenotype (63, 64). In GC, the accumulation of polymorphonuclear myeloid-derived suppressor cells inhibits glycolysis in CD8^+^ T cells, contributing to their depletion and facilitating immunotolerance (65). Additionally, the CD155/TIGIT (T-cell immunoreceptor with immunoglobulin and ITIM domains) signaling inhibits CD8^+^ T cell function, primarily through the suppression of their glucose utilization (66).

The heightened glycolytic activity within the TME leads to a significant accumulation of lactate, which causes acidification of the TME and adversely affects CD8^+^ T cell function (67). Lactate accumulation induces apoptosis in immune cells, further diminishing the efficacy of CD8^+^ T cells and NK cells (68). Moreover, lactate directly interacts with the histone lysine lactylation (Kla) site, stimulating gene expression in immune cells (69). The transition of M1-macrophages to the M2 phenotype may be linked to histone lactylation at the Kla site, highlighting a potential mechanism through which lactate accumulation contributes to immune tolerance (69). Furthermore, overexpression of the G protein-coupled receptor GPR81, a lactate receptor, in cancer cells has been associated with increased proliferation, drug resistance, and elevated PD-L1 expression (70–72). Activation of GPR81 on immune cells by lactate may also promote tumor growth in a paracrine manner (73). Notably, silencing GPR81 in mouse models revealed a suppression of Tregs production (74). Targeting glucose transporter protein 1 (GLUT1) has a promotive effect on the differentiation of CD8^+^ T cells into effector cells, and suppression of GLUT1 not only affects tumor metabolism but also induces the accumulation of reactive oxygen species (ROS), which mediates tumor cell apoptosis through activation of the TNF-α signaling pathway (75, 76). The checkpoint blockade antibodies against CTLA-4, PD-1, and PD-L1, can restore glucose in tumor microenvironment, permitting T cell glycolysis and IFN-γ production (77). Collectively, the multifaceted role of lactate in the TME underscores its potential as a therapeutic target for GC treatment, particularly those aimed at enhancing anti-tumor immunity.

### 3.2 Glutamine in TME

Glutamine serves as an essential nutrient for both immune cells and tumor cells. In the TME, tumor cells exhibit a heightened demand for glutamine to fuel their rapid growth and proliferation, making the metabolism of glutamine a key area of interest in cancer research (78–80). Elevated glutamine uptake has been associated with poorer prognoses in cancer patients, as its abundance supports the metabolic reprogramming that facilitates tumor progression (81). In GC, overexpression of the sodium-coupled neutral amino acid transporter 2 has been shown to increase intracellular glutamine levels, and decreased ROS production. This reduction promotes the stemness and survival of GC cells, thereby enhancing therapeutic resistance and driving tumor aggressiveness (82). Conversely, reduced glutamine in cell cultures were found to attenuate the stemness of GC cells, indicating a direct relationship between glutamine availability and tumor cell plasticity (82). Glutamine transferase 2 (TGM2) is a key enzyme in glutamine metabolism. Macrophages expressing TGM2 activate the NF-κB and ERK1/2 pathways, leading to the secretion of IL-1β, CSF-1, and MMPs, which contributes to the malignant development of GC (83, 84). In addition, glutamine metabolism has been found to drive the polarization of macrophages toward M2-phenotype, contributing to immune evasion and tumor progression (85). A transcription factor Myc, in concert with transaminases, drives a substantial increase in glutamine uptake in cancer cells (86, 87). This excessive glutamine consumption by GC tumor cells results in systemic glutamine depletion (88), which results in promotion of inflammatory differentiation in CD4^+^ T cells and the simultaneous suppression of T cell proliferation and activation (80, 89).

This polarization hampers the anti-tumor immune response and creates a favorable environment for tumor growth. Understanding the dual role of glutamine in supporting both tumor cell proliferation and immune suppression provides a compelling rationale for developing therapeutic strategies targeting glutamine metabolism in gastric cancer. By inhibiting key components of glutamine uptake and utilization, it may be possible to disrupt the metabolic flexibility of cancer cells and restore immune function, offering new avenues for therapeutic intervention.

### 3.3 Lipids in TME

Fatty acid (FA) metabolism in GC is heavily reliant on key mediators such as fatty acid translocase CD36 and carnitine palmitoyltransferase, which play pivotal roles in tumor progression and immune modulation within the TME (90). It has been found that co-culturing cancer cells with adipocytes leads to increased lipolysis in the adipocytes and promotes the release of fatty acids, which are then taken up by the tumor cells (91). In addition, lipoprotein lipase hydrolyzes lipids from dietary sources and subsequently CD36 takes up the fatty acids, and increased expression of lipoprotein lipase is found in a variety of cancers (92–94). Accumulation of FA induces the overexpression of CD36 and oxidized low-density lipoprotein (ox-LDL), triggering a cascade of deleterious effects. Specifically, CD36-dependent promotion of P38 phosphorylation leads to lipid peroxidation and ferroptosis, impairing the cytotoxic function of CD8^+^ tumor-infiltrating lymphocytes (TILs) and effectively suppressing the antitumor immune response (95). Moreover, the overexpression of CD36 interacts with apolipoprotein C-II (APOC2), which drives EMT and activates the PI3K-AKT-mTOR pathway. This activation by APOC2 enhances FA uptake in GC cells, further contributing to the immunosuppressive microenvironment and facilitating metastasis (96–98). In addition, TILs in GC, particularly the CD69^+^CD103^+^ tissue-resident memory (Trm) cells which are crucial for sustained immune surveillance, rely heavily on fatty acid oxidation. However, the aggressive lipid uptake by GC cells outcompetes Trm cells, leading to their depletion within the TME (99). The loss of Trm cells, results in a further decline in immune-mediated tumor control and contributes to immune tolerance (99). Further complicating the metabolic dynamics within the TME is the occurrence of mutations in ras homolog family member A (RHOA) in certain gastric cancer cells. These mutations activate the PI3K-AKT-mTOR signaling pathway, leading to elevated levels of free fatty acids (100). Tregs have a higher affinity for free fatty acid uptake compared to CD8^+^ T cells. This preferential uptake by Tregs promotes their accumulation within TME, exacerbating the immunosuppressive milieu and further hindering the natural immune defenses against the cancer (100).

Targeting simultaneously the three pathways—glycolysis, glutamine and lipid metabolism—represents promising therapeutic strategies in GC treatment. Future research should focus on elucidating the intricate metabolic interdependencies within the TME to identify optimal intervention points. Because lactate accumulation promotes fatty acid oxidation, while lipid oxidation in turn supports tumor cell glycolysis, and glutamine metabolism can feed lipid synthesis, a sequential inhibition strategy might be a potential approach. Initial targeting glycolysis to reduce lactate accumulation and alleviate tumor-induced acidification could create a more favorable immune milieu, enhancing the efficacy of immune-based therapies. Following this, the inhibition of glutamine and lipid metabolism could effectively disrupt compensatory metabolic pathways, further impairing tumor cell survival and proliferation.

### 3.4 Cytokines in TME

Cytokines are critical regulators in the complex orchestration of tumor immune tolerance, influencing the immune system’s ability to recognize and eliminate malignant cells. Mesenchymal stem cells (MSCs) within the TME secrete interleukin-15 (IL-15), which has been shown to increase Tregs and upregulate PD-L1, ultimately contributing to immune tolerance (101). TGF-β1, secreted by GC cells, also induces dysfunction in CD8^+^ T cells. Combined blockade of programmed death-1 (PD-1) and TGF-β1 effectively restores the functionality of CD8^+^ T cells, underscoring the potential therapeutic value of targeting these pathways in order to reinvigorate anti-tumor immune responses (102). Additionally, M2 macrophages secrete a variety of cytokines, including TGF-β1, which further suppresses the anti-tumor activity of NK cells (103). Moreover, the secretion of chitinase 3-like protein 1 by M2 macrophages, in conjunction with IL-13 receptor α2, has also been implicated in the suppression of NK cell activity (104). The chemokine CXCL8, produced by M2 macrophages, has been found to elevate PD-L1 levels, thereby enhancing the immunosuppressive potential of the TME (105). Concurrently, cytokines such as TNF-α and IL-6 activate the NF-κB and STAT3 signaling pathways in GC cells, leading to upregulation of PD-L1 and facilitating the immune escape of malignant cells (106). TGF-β, IL-10, and IFN-γ secreted by MDSC induce elevated levels of Tregs, which in turn enhance MDSC function via TGF-β and IL-35 (33). Notably, both TGF-β and IL-6 are also implicated in driving the polarization of neutrophils toward the immunosuppressive N2 phenotype, further complicating the immune landscape within the TME (46, 107).

In summary, TGF-β1, IL-10 and IL-35 act directly on immune cells, leading to either suppression of anti-tumor immune cells or enhancement of immunosuppressive cells; whereas IL-6, TNF-α and CXCL8 play important roles in the activation of immune checkpoints, which is important for immunotherapy tolerance. Future research aimed at disrupting these cytokine-mediated pathways may offer novel strategies for enhancing anti-tumor immunity and improving clinical outcomes for patients with GC. Multi-target combination therapies could be applied in GC treatment, for example, TGF-β blockers in combination with PD-1/PD-L1 inhibitors, or sequential therapies, such as blocking TGF-β or IL-6 first to release the immunosuppressive microenvironment, followed by the administration of immune checkpoint inhibitors.

### 3.5 Noncoding RNAs in TME

Non-coding RNAs (ncRNAs) represent a diverse class of RNA molecules that do not encode proteins but play critical roles in regulating gene expression and protein functionality. These molecules form intricate regulatory networks that significantly contribute to the development of tumor immune tolerance. Recent studies have elucidated the role of Linc00665 in TAM polarization. Specifically, Linc00665 activates the transcription factor BTB domain and CNC homology 1 (BACH1) and the activated BACH1 subsequently binds to the promoter of Wnt1, driving M2 polarization of macrophages (108). Similarly, lncRNA HCG18 promotes M2-type macrophage formation by downregulating miR-875-3p in macrophages, further reinforcing the immunosuppressive environment (109). And the lncRNA ANCR influences the polarization of M1-macrophages, resulting in a diminished M1 population, which facilitates GC cell invasion and metastasis (110).

LncRNA Linc0015 is markedly upregulated inn GC and negatively correlates with CD8^+^ T cell levels. Linc0015 impedes CD8^+^ T cell trafficking by interacting with the zeste homolog enhancer 2 and inhibiting the Cys-X-Cys ligand 9 and Cys-X-Cys ligand10/C-X-C motif chemokine receptor 3 axis, thereby contributing to immune evasion (111). In contrast, microRNA-105-5p exhibits low expression levels in gastric cancer; its overexpression enhances the activation of CD8^+^ T cells, thereby potentially restoring anti-tumor immunity (112). Furthermore, low levels of miR-128-3p have been associated with increased GC growth, while its overexpression reduces Tregs infiltration in GC tissues by reducing IL-16 (113). Additionally, microRNA-1290 mediates immunosuppression by inhibiting T cell proliferation through the granule head-like 2/zinc finger E-box binding homeobox 1/PD-L1 signaling axis (114).

## 4 Therapies against immune tolerance in gastric cancer

**Figure 2:**
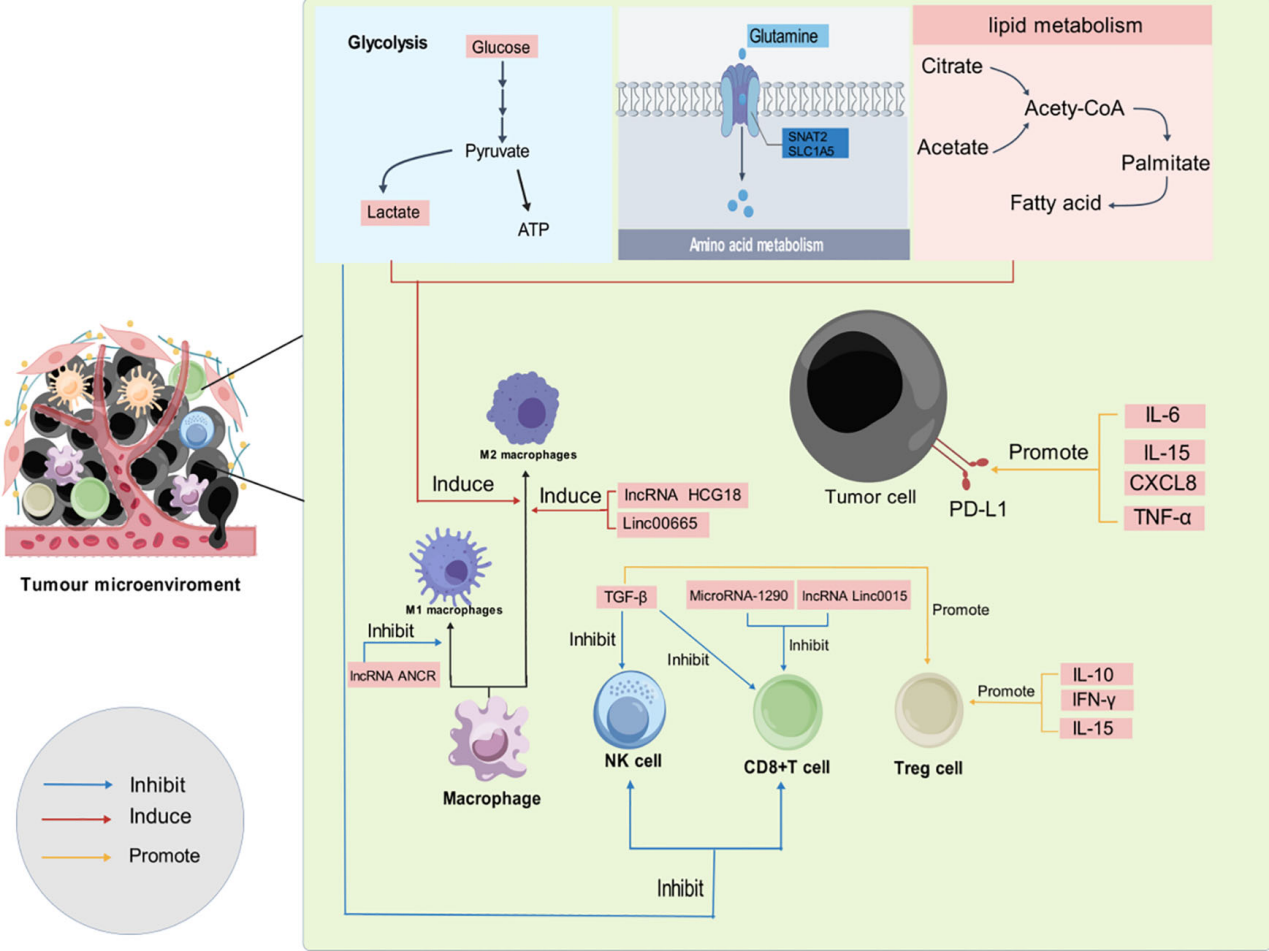
Development of immunosuppressive TME through various mechanisms. As the tumor progresses, an immunosuppressive TME is established through a variety of mechanisms, including glycolytic metabolism, glutamine metabolism, lipid metabolism, cytokine activity, and noncoding RNAs. (blue arrows indicate inhibition, red arrows indicate induction, and yellow arrows indicate promotion.).

A comprehensive understanding of the TME has significantly advanced therapy for GC. This enhanced knowledge allows for the identification of specific molecular and cellular interactions within the TME that can be targeted to enhance therapeutic efficacy (**Figure 2**). Immunotherapy offers distinct advantages over traditional treatment modalities, primarily due to its capacity to reinvigorate the functionality of immune cells, enabling them to effectively recognize and eradicate tumor cells and tends to exhibit a more favorable toxicity profile compared to conventional therapies, which often involve systemic cytotoxic agents. Currently, various immunotherapeutic strategies have been integrated into clinical practice for the management of gastric cancer (**Table 1**).

**Table 1:** Efficacy and limitations of various current immunotherapies.

Treatments	Efficacy	Limitations
**Immune Checkpoint** **Inhibitors**	1. Activate immune cells and alleviate immunosuppressive tumor microenvironment. 2. PD-1 inhibitors combined with chemotherapy significantly prolong survival. 3. PD-L1 inhibitors exhibit strong anti-tumor activity.	1. Limited efficacy for HER2-negative patients. 2. severe side effects. 3. Variable responses among patients necessitate biomarker identification.
**CAR-T Cell Therapy**	1. CAR-T cells targeting HER2, ICAM-1, etc., show significant efficacy in GC. 2. Novel CAR-T approaches like CDH17CART effectively treat gastrointestinal tumors. 3. CLDN18.2-specific CAR-T cells demonstrate robust tumor elimination with favorable safety.	1. Off-target effects. 2. TME factors suppress CAR-T function. 3. High costs and cell stability issues limit broader application.
**Biological Agents**	1. G47Δ virus reduces M2 macrophages and increases M1 macrophages and NK cells. 2. IFN-α2b hydrogels enhance T-cell migration and immune response. 3. DC modification promote DC maturation and induce T cell stimulation.	1. Lack of comprehensive human studies; efficacy and safety evidence are limited. 2. Clinical application is constrained; large-scale trials are required.
**Antibody-Drug Conjugates (ADCs)**	1. HER2-targeted ADCs show good efficacy in HER2-positive GC. 2. Novel ADCs (e.g., disitamab vedotin) demonstrate strong antitumor activity with controlled safety.	1. limited efficacy in HER2-negative cases. 2. Long-term safety and efficacy need further investigation.
**Other Treatments**	1. Tranilast enhances CD8^+^ T cell infiltration and reduces M2 macrophages. 2. Futibatinib demonstrates anti-tumor efficacy in various malignancies, including GC. 3. Metformin modulates CAFs to inhibit tumor progression.	1. Clinical application in GC requires optimization. 2. Potential toxicity risks need further evaluation.

### 4.1 Immune checkpoint inhibitors

PD-1 and PD-L1 inhibitors can effectively activate immune cells and release the immunosuppressive state in the TME of GC. PD-1 is a critical co-inhibitory receptor predominantly expressed on the surface of immune cells, where it plays an essential role in modulating immune responses. PD-1 interacts with its ligand PD-L1, leading to the suppression of anti-tumor immune responses (112, 115). PD-L1 is frequently overexpressed in various malignancies, including GC, where it contributes to the immune evasion of tumor cells (116). Navulizumab, a PD-1 inhibitor, has demonstrated significant efficacy in enhancing patient survival outcomes in clinical settings (117). Notably, the combination of navulizumab with conventional chemotherapy has resulted in markedly prolonged survival compared to chemotherapy alone, thereby representing a promising therapeutic strategy for advanced GC (8). Additionally, the PD-1 inhibitor camrelizumab, when combined with concurrent radiotherapy, has exhibited superior efficacy in treating GC patients (118). Pembrolizumab, another PD-L1 inhibitor, has exhibited strong anti-tumor activity in GC; however, attention must be paid to its side effects, which may affect patients’ quality of life (119). In contrast, avelumab, while demonstrating a more favorable safety profile, has not achieved outstanding efficacy compared to other PD-L1 inhibitors (120). A randomized, multicenter, double-blind phase 3 trial exploring the efficacy and safety of adjuvant nivolumab plus chemotherapy after gastrectomy with D2 or more extensive lymph-node dissection did not support the use of nivolumab for GC in postoperative adjuvant therapy (121). However, another phase 3 trial demonstrated a clinically meaningful improvement in median OS with nivolumab plus chemotherapy versus chemotherapy (14.3 vs. 10.2 months) for patients with non-HER2-positive GC (122). A phase 2 trial evaluating the combination of cabozantinib and pembrolizumab for the treatment of ICI-refractory or drug-resistant metastatic gastroesophageal adenocarcinoma found a 6-month progression-free survival of 22.2%, which is more than 4-fold higher than previous studies, demonstrating superior clinical benefit with a manageable safety profile (123).

Cytotoxic T lymphocyte-associated protein-4 (CTLA-4) represents another critical checkpoint molecule predominantly expressed on Tregs and activated T cells (124, 125). However, Ipilimumab and tremelimumab, CTLA-4 inhibitors, have not shown superior efficacy compared to standard supportive therapy in GC patients (126, 127). In addition to the conventional PD-1, PD-L1 and CTLA-4, there are now antibody therapies targeting other sites. *In vitro* investigations revealed that the administration of CD137 antibodies activates the NF-κB signaling pathway and enhances CD8^+^ T cell activity (128). Transfection of the MG-7 antigen (MG-7Ag) into DCs via viral vectors has demonstrated significant induction of anti-tumor effects through specific cytotoxic T lymphocytes (129).

Although ICIs marks a significant advancement in GC therapy, it is essential to acknowledge that these agents do not yield effective responses in all patients. ICIs are an effective option for HER2-positive GC patients, but their effectiveness for HER2-negative patients is still lacking, and more clinical trials are needed to prove their effectiveness and safety. Specific predictive biomarkers are needed to identify and segment patient subgroups that may benefit from the treatment. The variability in treatment efficacy, coupled with potential toxicities, underscores the necessity for ongoing research to identify predictive biomarkers for response, optimize treatment regimens, and expand the therapeutic options available for patients with GC.

### 4.2 Chimeric antigen receptor T cell

Chimeric antigen receptor T-cell (CAR-T) therapy represents a groundbreaking advancement in tumor immunotherapy, leveraging genetic engineering to enhance the anti-tumor capabilities of T cells. The process begins with the isolation of T cells from patients, followed by modification using viral vectors to introduce chimeric antigen receptors (CARs). This modification enables the engineered T cells to effectively recognize and bind to tumor-associated antigens (TAAs), thereby enhancing their capacity to mount a robust anti-tumor immune response upon reinfusion into the patients (130–132). The overexpression of HER2 on GC cells has prompted the development of CAR-T therapies targeting HER2. These therapies have demonstrated a high affinity for HER2-expressing GC cells, facilitating targeted destruction of malignant cells in an MHC-independent manner (133). Additionally, CAR-T cells directed against intercellular adhesion molecule 1 (ICAM-1) have exhibited substantial therapeutic efficacy against both primary and metastatic gastric tumors (134). Moreover, novel CAR-T approaches, such as CDH17CART driven by the VHH1 nanoantibody targeting CDH17, have shown promise in treating gastrointestinal tumors while sparing normal epithelial cells, thus minimizing collateral damage (135). The mesothelin (MSLN) protein, prevalent in normal mesothelial tissues and markedly overexpressed in gastric cancers, has also been a target for CAR-T therapies. Studies indicate that anti-MSLN CAR-T cells are effective in treating GC, with enhanced efficacy observed through local peritumoral administration (136). Claudin 18.2 (CLDN18.2), a gastric-specific membrane protein present in approximately 70% of primary gastric adenocarcinomas and their metastases, has emerged as another target for CAR-T therapy (137). Notably, CAR-T cells specific for CLDN18.2 have demonstrated remarkable tumor elimination capabilities while maintaining a favorable safety profile, avoiding severe toxicity to surrounding normal tissues (138).

CAR-T cell therapy faces several challenges that can compromise its efficacy, particularly in the context of solid tumors. One significant limitation is the off-target effects stemming from the targeting of tumor-associated antigens, that may also be present on non-tumor tissues, leading to unintended toxicity (139). Furthermore, the TME, enriched with factors such as TGF-β, IL-4, and IL-10, can significantly dampen the functionality of CAR-T cells, impeding their antitumor activity (140). Another major concern is the potential for severe cytotoxic effects associated with CAR-T cell therapy, most notably cytokine release syndrome. Cytokine release syndrome characterized by fever, hypotension, hypoxia, and multi-organ dysfunction due to elevated inflammatory cytokines, poses a substantial clinical risk. In severe cases, this can escalate to immune effector cell-associated neurotoxicity syndrome, further complicating patient management (141). To overcome these challenges, several innovative strategies have been proposed. Addressing the lack of tumor-specific antigens in solid tumors, one promising approach involves engineering T cells to express multiple CARs or multispecific CARs. These T cells are designed to activate only when multiple target antigens are simultaneously expressed on tumor cells, thereby minimizing off-target effects and enhancing tumor specificity (142–144). Additionally, genetic modifications, such as the knockdown of TGF-β signaling within CAR-T cells, have been shown to augment their antitumor efficacy by mitigating the suppressive influences of the TME (145).

CAR-T cells, with their engineered specificity, can effectively counteract the immunosuppressive state, thereby achieving therapeutic results. However, challenges remain, particularly regarding the high costs associated with T-cell therapy, as well as concerns about the stability and proliferation of the modified T cells. Continued research is essential to address these challenges and optimize CAR-T therapies for broader clinical application and improvement of patient outcomes in GC.

### 4.3 Biological agents

*In vivo* experiments involving the construction of relaxin-carrying lyssaviruses have demonstrated their efficacy in degrading the ECM of GC, facilitating the infiltration of activated T cells into the TME and thereby exerting significant antitumor effects (146). G47Δ, a third-generation oncolytic herpes simplex virus type 1, has shown promising results when administered via intratumoral injection into GC tumors, leading to a notable reduction in M2-macrophages coupled with an increase in M1-macrophages and NK cells, thereby enhancing the therapeutic potential against GC (147). Furthermore, Yan et al. successfully modulated the immunosuppressive milieu by co-loading injectable shear-thinning hydrogels with polypodophyllin II (PP2) and resiquimod (R848). This innovative approach resulted in the repolarization of M2-macrophages to M1-macrophages and augmented infiltration of CD8^+^ T cells, reflecting a strategic shift in the immune response (148). The incorporation of IFN-α2b into hydroxypropyl cellulose hydrogels has been shown to maintain its activity over extended periods, allowing for stable release that stimulates T cells; when combined with low-dose radiation, this approach effectively promotes T cell migration into gastric cancer tissues, thereby enhancing the immunotherapeutic effect (149). Furthermore, constructing a recombinant adenovirus carrying the secondary lymphoid tissue chemokine gene for DC modification has been found to substantially promote DC maturation and enhance their ability to induce T cell chemotaxis and stimulation, leading to a robust cancer immune response in GC (150). Lastly, in a mouse model, the fusion protein dsNKG2D-IL-15 was identified as a potent agent capable of recruiting and activating NK cells, thereby exerting powerful immune effects against tumor cells (151).

In summary, compared to traditional immunotherapy, biological agents offer a distinct and highly advantageous mechanism by directly targeting the patient’s immune cells. These agents are designed to alleviate the immunosuppressive state by restoring the functional capacity of immune cells, thereby effectively inhibiting tumor progression. This precision-targeted approach not only amplifies antitumor immunity but also holds the potential for improved safety profiles, as it minimizes off-target effects and systemic toxicity, providing a more secure therapeutic option for patients. Nevertheless, the clinical application of biological agents remains constrained by a relative paucity of comprehensive studies evaluating the efficacy and safety in humans. Rigorous, large-scale clinical trials are imperative to substantiate their therapeutic potential and establish evidence-based protocols for their integration into cancer treatment paradigms.

### 4.4 Antibody-drug conjugates

ADCs consist of drugs cross-linked with monoclonal antibodies against tumor cell antigens that are expressed highly in tumor cells, and thus ADCs provide cytotoxic drugs to specifically recognize tumor cells, thereby improving clinical outcomes (152).

A non-randomized, open-label, multi-dose Phase 1 trial with trastuzumab -deruxtecan (DS-8201a), a HER2-targeting ADC, in HER2-positive GC patients showed a manageable safety profile and showed preliminary activity (153). Based on the results of the DESTINY-Gastric01 study, trastuzumab-deruxtecan is currently approved by the FDA for the treatment of adult patients with unresectable, locally advanced or metastatic GC who have received a prior trastuzumab-based regimen (154). Beyond trastuzumab-deruxtecan, disitamab vedotin, a novel anti-HER2 ADC, has shown significant promise. In a Phase I trial involving 57 patients with advanced HER2-positive GC, disitamab vedotin demonstrated a favorable safety profile and robust antitumor activity (155). In another open-label, multicenter, Phase 2 study, disitamab vedotin showed promising activity with a controlled safety profile at an ORR of 24.8%, validating its efficacy and safety in patients with advanced gastric or gastroesophageal conjugate cancer with HER2 overexpression (156). In addition, a multicenter, open-label, dose-escalation and extension Phase 1 trial demonstrated superior clinical efficacy and safety of disitamab vedotin in combination with teraplizumab (157).

### 4.5 Other treatments

Recent studies have highlighted the anti-allergic agent Tranilast as a promising candidate for enhancing CD8^+^ T cell infiltration while concurrently diminishing the prevalence of M2-macrophages, thereby contributing to the restoration of a more immunocompetent TME in GC (158). Similarly, Futibatinib, a selective inhibitor of fibroblast growth factor receptors 1 to 4 (FGFR1-4), has demonstrated notable anti-tumor efficacy across various malignancies, including GC (159). Moreover, Metformin has been identified as a modulator of GC progression through its ability to regulate the secretion of calmodulin-like protein 3 (Calml3) from CAFs (160). IPI549, a selective inhibitor of the PI3K-γ isoenzyme, has shown potential in restoring macrophage functionality, thereby enhancing the anti-tumor immune responses of T cells (161). In addition, *in vivo* experiments indicate that methionine enkephalin (MENK) exerts its effects by inhibiting the PI3K/AKT/mTOR signaling axis in GC cells, facilitating the polarization towards M1-macrophages (162).

While these agents have shown efficacy in GC treatment, it is noteworthy that most were originally developed for other therapeutic indications. This divergence poses significant challenges for their precise application in clinical settings, necessitating careful consideration of how to leverage their anti-tumor properties while minimizing potential toxicities. Thus, further investigation is warranted to optimize their clinical utilization in gastric cancer treatment.

Some herbal medicines possess potential for reshaping TME in GC. For instance, paeoniflorin can inhibit IL-6 secretion in CAFs and effectively ameliorate immunosuppression within TME in GC (160). However, the clinical application of herbal medicine in cancer therapy is limited due to the lack of evidence from randomized controlled trials. To integrate herbal medicine into mainstream therapies, it is essential to identify, isolate and standardize of the active ingredients in herbs. This ensures consistency and reproducibility in the clinical setting.

## 5 Conclusion

Currently, the development of therapeutic strategies that target and modify the immunosuppressive TME is indeed a critical frontier in the treatment of GC, as well as other solid tumors. Reversing the immunosuppressive environment is a pivotal strategy in cancer immunotherapy. This approach aims to enhance the activity of effector immune cells, such as cytotoxic CD8^+^ T cells and NK cells, thereby improving their ability to recognize and eliminate tumor cells. Emerging therapeutic modalities, such as immune checkpoint inhibitors, cytokine modulation, adoptive cell therapies, and novel biological agents targeting metabolic and signaling pathways within the TME, are poised to revolutionize our approach to GC treatment. To achieve this breakthrough, it is essential to have an integrative understanding of the molecular and cellular mechanisms that drive immune dysfunction in GC. Additionally, designing combinatorial therapies that synergistically target the immunosuppressive TME is crucial. By restoring immune homeostasis and enhancing antitumor immunity, these advancements hold the potential to significantly improve clinical outcomes and redefine the therapeutic landscape for gastric cancer.

## References

[1] Sung H Ferlay J Siegel RL Laversanne M Soerjomataram I Jemal A. Global cancer statistics 2020: GLOBOCAN estimates of incidence and mortality worldwide for 36 cancers in 185 countries. CA Cancer J Clin. (2021) 71:209–49. doi: 10.3322/caac.21660 33538338

[2] Biondi A Persiani R Cananzi F Zoccali M Vigorita V Tufo A. R0 resection in the treatment of gastric cancer: room for improvement. World J Gastroenterol. (2010) 16:3358–70. doi: 10.3748/wjg.v16.i27.3358 PMC2904881 20632437

[3] Cunningham D Starling N Rao S Iveson T Nicolson M Coxon F. Capecitabine and oxaliplatin for advanced esophagogastric cancer. N Engl J Med. (2008) 358:36–46. doi: 10.1056/NEJMoa073149 18172173

[4] Park JS Lim JY Park SK Kim MK Ko HS Yoon SO. Prognostic factors of second and third line chemotherapy using 5-fu with platinum, irinotecan, and taxane for advanced gastric cancer. Cancer Res Treat. (2011) 43:236–43. doi: 10.4143/crt.2011.43.4.236 PMC3253866 22247709

[5] Bang YJ Van Cutsem E Feyereislova A Chung HC Shen L Sawaki A. Trastuzumab in combination with chemotherapy versus chemotherapy alone for treatment of HER2-positive advanced gastric or gastro-oesophageal junction cancer (ToGA): a phase 3, open-label, randomised controlled trial. Lancet. (2010) 376:687–97. doi: 10.1016/S0140-6736(10)61121-X 20728210

[6] Ishimine Y Goto A Watanabe Y Yajima H Nakagaki S Yabana T. Loss of HER2 positivity after trastuzumab in HER2-positive gastric cancer: is change in HER2 status significantly frequent? Case Rep Gastrointest Med. (2015) 2015:132030. doi: 10.1155/2015/132030 25893119 PMC4393931

[7] Van Cutsem E Bang YJ Feng-Yi F Xu JM Lee KW Jiao SC. HER2 screening data from ToGA: targeting HER2 in gastric and gastroesophageal junction cancer. Gastric Cancer. (2015) 18:476–84. doi: 10.1007/s10120-014-0402-y PMC4511072 25038874

[8] Janjigian YY Shitara K Moehler M Garrido M Salman P Shen L. First-line nivolumab plus chemotherapy versus chemotherapy alone for advanced gastric, gastro-oesophageal junction, and oesophageal adenocarcinoma (CheckMate 649): a randomised, open-label, phase 3 trial. Lancet. (2021) 398:27–40. doi: 10.1016/S0140-6736(21)00797-2 34102137 PMC8436782

[9] Gajewski TF Schreiber H Fu YX. Innate and adaptive immune cells in the tumor microenvironment. Nat Immunol. (2013) 14:1014–22. doi: 10.1038/ni.2703 PMC4118725 24048123

[10] Marvel D Gabrilovich DI. Myeloid-derived suppressor cells in the tumor microenvironment: expect the unexpected. J Clin Invest. (2015) 125:3356–64. doi: 10.1172/JCI80005 PMC4588239 26168215

[11] Fakharian F Asgari B Nabavi-Rad A Sadeghi A Soleimani N Yadegar A. The interplay between Helicobacter pylori and the gut microbiota: An emerging driver influencing the immune system homeostasis and gastric carcinogenesis. Front Cell Infect Microbiol. (2022) 12:953718. doi: 10.3389/fcimb.2022.953718 36046747 PMC9423097

[12] Yu X Ou J Wang L Li Z Ren Y Xie L. Gut microbiota modulate CD8(+) T cell immunity in gastric cancer through Butyrate/GPR109A/HOPX. Gut Microbes. (2024) 16:2307542. doi: 10.1080/19490976.2024.2307542 38319728 PMC10854374

[13] Chen D Zhang X Li Z Zhu B. Metabolic regulatory crosstalk between tumor microenvironment and tumor-associated macrophages. Theranostics. (2021) 11:1016–30. doi: 10.7150/thno.51777 PMC7738889 33391518

[14] Mantovani A Marchesi F Malesci A Laghi L Allavena P. Tumour-associated macrophages as treatment targets in oncology. Nat Rev Clin Oncol. (2017) 14:399–416. doi: 10.1038/nrclinonc.2016.217 28117416 PMC5480600

[15] Petersen CP Meyer AR De Salvo C Choi E Schlegel C Petersen A. A signalling cascade of IL-33 to IL-13 regulates metaplasia in the mouse stomach. Gut. (2018) 67:805–17. doi: 10.1136/gutjnl-2016-312779 PMC5681443 28196875

[16] Biswas SK Mantovani A. Macrophage plasticity and interaction with lymphocyte subsets: cancer as a paradigm. Nat Immunol. (2010) 11:889–96. doi: 10.1038/ni.1937 20856220

[17] Cui X Qin T Zhao Z Yang G Sanches JGP Zhang Q. Pentraxin-3 inhibits milky spots metastasis of gastric cancer by inhibiting M2 macrophage polarization. J Cancer. (2021) 12:4686–97. doi: 10.7150/jca.58698 PMC8210545 34149932

[18] Tang C Lei X Xiong L Hu Z Tang B. HMGA1B/2 transcriptionally activated-POU1F1 facilitates gastric carcinoma metastasis via CXCL12/CXCR4 axis-mediated macrophage polarization. Cell Death Dis. (2021) 12:422. doi: 10.1038/s41419-021-03703-x 33927188 PMC8084942

[19] Xia Y Rao L Yao H Wang Z Ning P. Engineering macrophages for cancer immunotherapy and drug delivery. Adv Mater. (2020) 32:2002054. doi: 10.1002/adma.202002054 32856350

[20] A phase 1, first in human study of adenovirally transduced autologous macrophages engineered to contain an anti-HER2 chimeric antigen receptor in subjects with HER2 overexpressing solid tumors. (2020).

[21] Kawazoe A Fukuoka S Nakamura Y Kuboki Y Wakabayashi M Nomura S. Lenvatinib plus pembrolizumab in patients with advanced gastric cancer in the first-line or second-line setting (EPOC1706): an open-label, single-arm, phase 2 trial. Lancet Oncol. (2020) 21:1057–65. doi: 10.1016/S1470-2045(20)30271-0 32589866

[22] Rannikko JH Verlingue L Miguel M Pasanen A Robbrecht D Skytta T. Bexmarilimab-induced macrophage activation leads to treatment benefit in solid tumors: The phase I/II first-in-human MATINS trial. Cell Rep Med. (2023) 4:101307. doi: 10.1016/j.xcrm.2023.101307 38056464 PMC10772343

[23] Li J Bai Y Chen Z Ying J Guo Y Fang W. SAFFRON-104: a phase Ib/II study of sitravatinib alone or with tislelizumab in advanced hepatocellular carcinoma and gastric cancer/gastroesophageal junction cancer. Cancer Immunol Immunother. (2024) 73:219. doi: 10.1007/s00262-024-03806-2 39235596 PMC11377389

[24] Merck S Dohme LLC eds. A Phase 1/2, Open-Label, Dose Escalation and Expansion Study with PT886 Followed by a Multi-cohorT Study in Patients with Advanced GastrIc, Gastroesophageal JuNction (GEJ), or Pancreatic Ductal AdEnocarcinomas of PT886, in Combination with Either ChemotherApy, And/or the ChecKpoint Inhibitor Pembrolizumab. the TWINPEAK Study (2022).

[25] Wherry EJ Ahmed R. Memory CD8 T-cell differentiation during viral infection. J Virol. (2004) 78:5535–45. doi: 10.1128/JVI.78.11.5535-5545.2004 PMC415833 15140950

[26] Farhood B Najafi M Mortezaee K. CD8(+) cytotoxic T lymphocytes in cancer immunotherapy: A review. J Cell Physiol. (2019) 234:8509–21. doi: 10.1002/jcp.v234.6 30520029

[27] Xu J Guo R Jia J He Y He S. Activation of Toll-like receptor 2 enhances peripheral and tumor-infiltrating CD8(+) T cell cytotoxicity in patients with gastric cancer. BMC Immunol. (2021) 22:67. doi: 10.1186/s12865-021-00459-z 34620075 PMC8499526

[28] Wei X Zhang J Gu Q Huang M Zhang W Guo J. Reciprocal expression of IL-35 and IL-10 defines two distinct effector treg subsets that are required for maintenance of immune tolerance. Cell Rep. (2017) 21:1853–69. doi: 10.1016/j.celrep.2017.10.090 29141218

[29] Budhu S Schaer DA Li Y Toledo-Crow R Panageas K Yang X. Blockade of surface-bound TGF-β on regulatory T cells abrogates suppression of effector T cell function in the tumor microenvironment. Sci Signal. (2017) 10. doi: 10.1126/scisignal.aak9702 PMC5851440 28851824

[30] Liu C Chikina M Deshpande R Menk AV Wang T Tabib T. Treg cells promote the SREBP1-dependent metabolic fitness of tumor-promoting macrophages via repression of CD8(+) T cell-derived interferon-γ. Immunity. (2019) 51:381–397.e6. doi: 10.1016/j.immuni.2019.06.017 31350177 PMC6703933

[31] Wang J Zhao X Wan YY. Intricacies of TGF-β signaling in Treg and Th17 cell biology. Cell Mol Immunol. (2023) 20:1002–22. doi: 10.1038/s41423-023-01036-7 PMC10468540 37217798

[32] Huard B Mastrangeli R Prigent P Bruniquel D Donini S El-Tayar N. Characterization of the major histocompatibility complex class II binding site on LAG-3 protein. Proc Natl Acad Sci U.S.A. (1997) 94:5744–9. doi: 10.1073/pnas.94.11.5744 PMC20850 9159144

[33] Li C Jiang P Wei S Xu X Wang J. Regulatory T cells in tumor microenvironment: new mechanisms, potential therapeutic strategies and future prospects. Mol Cancer. (2020) 19:116. doi: 10.1186/s12943-020-01234-1 32680511 PMC7367382

[34] Rocha S Basto AP Ijsselsteijn ME Teles SP Azevedo MM Gonçalves G. Immunophenotype of gastric tumors unveils a pleiotropic role of regulatory T cells in tumor development. Cancers (Basel). (2021) 13:421. doi: 10.3390/cancers13030421 33498681 PMC7865950

[35] Qu Y Wang X Bai S Niu L Zhao G Yao Y. The effects of TNF-α/TNFR2 in regulatory T cells on the microenvironment and progression of gastric cancer. Int J Cancer. (2022) 150:1373–91. doi: 10.1002/ijc.v150.8 PMC9298834 34766338

[36] Cerwenka A Lanier LL. Natural killers join the fight against cancer. Science. (2018) 359:1460–1. doi: 10.1126/science.aat2184 29599226

[37] Saito H Takaya S Osaki T Ikeguchi M. Increased apoptosis and elevated Fas expression in circulating natural killer cells in gastric cancer patients. Gastric Cancer. (2013) 16:473–9. doi: 10.1007/s10120-012-0210-1 23179366

[38] Mimura K Kamiya T Shiraishi K Kua LF Shabbir A So J. Therapeutic potential of highly cytotoxic natural killer cells for gastric cancer. Int J Cancer. (2014) 135:1390–8. doi: 10.1002/ijc.v135.6 24615495

[39] Szkaradkiewicz A Karpiński TM Drews M Borejsza-Wysocki M Majewski P Andrzejewska E. Natural killer cell cytotoxicity and immunosuppressive cytokines (IL-10, TGF-beta1) in patients with gastric cancer. J BioMed Biotechnol. (2010) 2010:901564. doi: 10.1155/2010/901564 20445748 PMC2860365

[40] Li T Zhang Q Jiang Y Yu J Hu Y Mou T. Gastric cancer cells inhibit natural killer cell proliferation and induce apoptosis via prostaglandin E2. Oncoimmunology. (2016) 5:e1069936. doi: 10.1080/2162402X.2015.1069936 27057432 PMC4801461

[41] Keener AB. Natural killers: cataloging immune cells for immunotherapy. Nat Med. (2015) 21:207–8. doi: 10.1038/nm0315-207 25742450

[42] Gao X Mi Y Guo N Xu H Xu L Gou X. Cytokine-induced killer cells as pharmacological tools for cancer immunotherapy. Front Immunol. (2017) 8:774. doi: 10.3389/fimmu.2017.00774 28729866 PMC5498561

[43] Kono K Takahashi A Ichihara F Sugai H Fujii H Matsumoto Y. Impaired antibody-dependent cellular cytotoxicity mediated by herceptin in patients with gastric cancer. Cancer Res. (2002) 62:5813–7. 12384543

[44] Catenacci DVT Rasco D Lee J Rha SY Lee KW Bang YJ. Phase I escalation and expansion study of bemarituzumab (FPA144) in patients with advanced solid tumors and FGFR2b-selected gastroesophageal adenocarcinoma. J Clin Oncol. (2020) 38:2418–26. doi: 10.1200/JCO.19.01834 PMC7367551 32167861

[45] Uribe-Querol E Rosales C. Neutrophils in cancer: two sides of the same coin. J Immunol Res. (2015) 2015:983698. doi: 10.1155/2015/983698 26819959 PMC4706937

[46] Fridlender ZG Sun J Kim S Kapoor V Cheng G Ling L. Polarization of tumor-associated neutrophil phenotype by TGF-beta: “N1” versus “N2” TAN. Cancer Cell. (2009) 16:183–94. doi: 10.1016/j.ccr.2009.06.017 PMC2754404 19732719

[47] Antuamwine BB Bosnjakovic R Hofmann-Vega F Wang X Theodosiou T Iliopoulos I. N1 versus N2 and PMN-MDSC: A critical appraisal of current concepts on tumor-associated neutrophils and new directions for human oncology. Immunol Rev. (2023) 314:250–79. doi: 10.1111/imr.v314.1 36504274

[48] Zhao Y Rahmy S Liu Z Zhang C Lu X. Rational targeting of immunosuppressive neutrophils in cancer. Pharmacol Ther. (2020) 212:107556. doi: 10.1016/j.pharmthera.2020.107556 32343986

[49] Wang Y Zhai J Zhang T Han S Zhang Y Yao X. Tumor-associated neutrophils can predict lymph node metastasis in early gastric cancer. Front Oncol. (2020) 10:570113. doi: 10.3389/fonc.2020.570113 33072602 PMC7537418

[50] Mak TK Li X Huang H Wu K Huang Z He Y. The cancer-associated fibroblast-related signature predicts prognosis and indicates immune microenvironment infiltration in gastric cancer. Front Immunol. (2022) 13:951214. doi: 10.3389/fimmu.2022.951214 35967313 PMC9372353

[51] Li X Sun Z Peng G Xiao Y Guo J Wu B. Single-cell RNA sequencing reveals a pro-invasive cancer-associated fibroblast subgroup associated with poor clinical outcomes in patients with gastric cancer. Theranostics. (2022) 12:620–38. doi: 10.7150/thno.60540 PMC8692898 34976204

[52] Li D Xia L Huang P Wang Z Guo Q Huang C. Cancer-associated fibroblast-secreted IGFBP7 promotes gastric cancer by enhancing tumor associated macrophage infiltration via FGF2/FGFR1/PI3K/AKT axis. Cell Death Discovery. (2023) 9:17. doi: 10.1038/s41420-023-01336-x 36681667 PMC9867714

[53] Li Y Zheng Y Huang J Nie RC Wu QN Zuo Z. CAF-macrophage crosstalk in tumour microenvironments governs the response to immune checkpoint blockade in gastric cancer peritoneal metastases. Gut. (2024) 74(3):350–63. doi: 10.1136/gutjnl-2024-333617 PMC11874311 39537239

[54] Yao L Hou J Wu X Lu Y Jin Z Yu Z. Cancer-associated fibroblasts impair the cytotoxic function of NK cells in gastric cancer by inducing ferroptosis via iron regulation. Redox Biol. (2023) 67:102923. doi: 10.1016/j.redox.2023.102923 37832398 PMC10582581

[55] Mao X Xu J Wang W Liang C Hua J Liu J. Crosstalk between cancer-associated fibroblasts and immune cells in the tumor microenvironment: new findings and future perspectives. Mol Cancer. (2021) 20:131. doi: 10.1186/s12943-021-01428-1 34635121 PMC8504100

[56] Jang M Koh I Lee JE Lim JY Cheong JH Kim P. Increased extracellular matrix density disrupts E-cadherin/β-catenin complex in gastric cancer cells. Biomater Sci. (2018) 6:2704–13. doi: 10.1039/C8BM00843D 30151505

[57] Butler JM Kobayashi H Rafii S. Instructive role of the vascular niche in promoting tumour growth and tissue repair by angiocrine factors. Nat Rev Cancer. (2010) 10:138–46. doi: 10.1038/nrc2791 PMC2944775 20094048

[58] Georganaki M van Hooren L Dimberg A. Vascular targeting to increase the efficiency of immune checkpoint blockade in cancer. Front Immunol. (2018) 9:3081. doi: 10.3389/fimmu.2018.03081 30627131 PMC6309238

[59] Motz GT Santoro SP Wang LP Garrabrant T Lastra RR Hagemann IS. Tumor endothelium FasL establishes a selective immune barrier promoting tolerance in tumors. Nat Med. (2014) 20:607–15. doi: 10.1038/nm.3541 PMC4060245 24793239

[60] Vaupel P Schmidberger H Mayer A. The Warburg effect: essential part of metabolic reprogramming and central contributor to cancer progression. Int J Radiat Biol. (2019) 95:912–9. doi: 10.1080/09553002.2019.1589653 30822194

[61] Yuan LW Yamashita H Seto Y. Glucose metabolism in gastric cancer: The cutting-edge. World J Gastroenterol. (2016) 22:2046–59. doi: 10.3748/wjg.v22.i6.2046 PMC4726677 26877609

[62] Bader JE Voss K Rathmell JC. Targeting metabolism to improve the tumor microenvironment for cancer immunotherapy. Mol Cell. (2020) 78:1019–33. doi: 10.1016/j.molcel.2020.05.034 PMC7339967 32559423

[63] He Z Chen D Wu J Sui C Deng X Zhang P. Yes associated protein 1 promotes resistance to 5-fluorouracil in gastric cancer by regulating GLUT3-dependent glycometabolism reprogramming of tumor-associated macrophages. Arch Biochem Biophys. (2021) 702:108838. doi: 10.1016/j.abb.2021.108838 33727040

[64] Yao X He Z Qin C Deng X Bai L Li G. SLC2A3 promotes macrophage infiltration by glycolysis reprogramming in gastric cancer. Cancer Cell Int. (2020) 20:503. doi: 10.1186/s12935-020-01599-9 33061855 PMC7552479

[65] Zhou X Fang D Liu H Ou X Zhang C Zhao Z. PMN-MDSCs accumulation induced by CXCL1 promotes CD8(+) T cells exhaustion in gastric cancer. Cancer Lett. (2022) 532:215598. doi: 10.1016/j.canlet.2022.215598 35176418

[66] He W Zhang H Han F Chen X Lin R Wang W. CD155T/TIGIT signaling regulates CD8(+) T-cell metabolism and promotes tumor progression in human gastric cancer. Cancer Res. (2017) 77:6375–88. doi: 10.1158/0008-5472.CAN-17-0381 28883004

[67] Ippolito L Morandi A Giannoni E Chiarugi P. Lactate: A metabolic driver in the tumour landscape. Trends Biochem Sci. (2019) 44:153–66. doi: 10.1016/j.tibs.2018.10.011 30473428

[68] Brand A Singer K Koehl GE Kolitzus M Schoenhammer G Thiel A. LDHA-associated lactic acid production blunts tumor immunosurveillance by T and NK cells. Cell Metab. (2016) 24:657–71. doi: 10.1016/j.cmet.2016.08.011 27641098

[69] Zhang D Tang Z Huang H Zhou G Cui C Weng Y. Metabolic regulation of gene expression by histone lactylation. Nature. (2019) 574:575–80. doi: 10.1038/s41586-019-1678-1 PMC6818755 31645732

[70] Feng J Yang H Zhang Y Wei H Zhu Z Zhu B. Tumor cell-derived lactate induces TAZ-dependent upregulation of PD-L1 through GPR81 in human lung cancer cells. Oncogene. (2017) 36:5829–39. doi: 10.1038/onc.2017.188 28604752

[71] Wagner W Kania KD Blauz A Ciszewski WM. The lactate receptor (HCAR1/GPR81) contributes to doxorubicin chemoresistance via ABCB1 transporter up-regulation in human cervical cancer HeLa cells. J Physiol Pharmacol. (2017) 68:555–64. 29151072

[72] Lee YJ Shin KJ Park SA Park KS Park S Heo K. G-protein-coupled receptor 81 promotes a Malignant phenotype in breast cancer through angiogenic factor secretion. Oncotarget. (2016) 7:70898–911. doi: 10.18632/oncotarget.12286 PMC5342597 27765922

[73] Brown TP Bhattacharjee P Ramachandran S Sivaprakasam S Ristic B Sikder MOF. The lactate receptor GPR81 promotes breast cancer growth via a paracrine mechanism involving antigen-presenting cells in the tumor microenvironment. Oncogene. (2020) 39:3292–304. doi: 10.1038/s41388-020-1216-5 32071396

[74] Ranganathan P Shanmugam A Swafford D Suryawanshi A Bhattacharjee P Hussein MS. GPR81, a cell-surface receptor for lactate, regulates intestinal homeostasis and protects mice from experimental colitis. J Immunol. (2018) 200:1781–9. doi: 10.4049/jimmunol.1700604 PMC5858928 29386257

[75] Cao J Liao S Zeng F Liao Q Luo G Zhou Y. Effects of altered glycolysis levels on CD8+ T cell activation and function. Cell Death Dis. (2023) 14:407. doi: 10.1038/s41419-023-05937-3 37422501 PMC10329707

[76] Wu L Jin Y Zhao X Tang K Zhao Y Tong L. Tumor aerobic glycolysis confers immune evasion through modulating sensitivity to T&xa0;cell-mediated bystander killing via TNF-&x3b1. Cell Metab. (2023) 35:1580–1596.e9. doi: 10.1016/j.cmet.2023.07.001 37506695

[77] Chang CH Qiu J O'Sullivan D Buck MD Noguchi T Curtis JD. Metabolic competition in the tumor microenvironment is a driver of cancer progression. Cell. (2015) 162:1229–41. doi: 10.1016/j.cell.2015.08.016 PMC4864363 26321679

[78] Hensley CT Wasti AT DeBerardinis RJ. Glutamine and cancer: cell biology, physiology, and clinical opportunities. J Clin Invest. (2013) 123:3678–84. doi: 10.1172/JCI69600 PMC3754270 23999442

[79] Yang L Venneti S Nagrath D. Glutaminolysis: A hallmark of cancer metabolism. Annu Rev BioMed Eng. (2017) 19:163–94. doi: 10.1146/annurev-bioeng-071516-044546 28301735

[80] Carr EL Kelman A Wu GS Gopaul R Senkevitch E Aghvanyan A. Glutamine uptake and metabolism are coordinately regulated by ERK/MAPK during T lymphocyte activation. J Immunol. (2010) 185:1037–44. doi: 10.4049/jimmunol.0903586 PMC2897897 20554958

[81] Fu Q Xu L Wang Y Jiang Q Liu Z Zhang J. Tumor-associated macrophage-derived interleukin-23 interlinks kidney cancer glutamine addiction with immune evasion. Eur Urol. (2019) 75:752–63. doi: 10.1016/j.eururo.2018.09.030 30293904

[82] Nie K Cai M. SNAT2/SLC38A2 confers the stemness of gastric cancer cells via regulating glutamine level. Dig Dis Sci. (2022) 67:2948–56. doi: 10.1007/s10620-021-07110-2 34173116

[83] Wang X Yu Z Zhou Q Wu X Chen X Li J. Tissue transglutaminase-2 promotes gastric cancer progression via the ERK1/2 pathway. Oncotarget. (2016) 7:7066–79. doi: 10.18632/oncotarget.6883 PMC4872769 26771235

[84] Cho SY Oh Y Jeong EM Park S Lee D Wang X. Amplification of transglutaminase 2 enhances tumor-promoting inflammation in gastric cancers. Exp Mol Med. (2020) 52:854–64. doi: 10.1038/s12276-020-0444-7 PMC7272405 32467608

[85] Hu X Ma Z Xu B Li S Yao Z Liang B. Glutamine metabolic microenvironment drives M2 macrophage polarization to mediate trastuzumab resistance in HER2-positive gastric cancer. Cancer Commun (Lond). (2023) 43:909–37. doi: 10.1002/cac2.12459 PMC10397568 37434399

[86] Ye J Huang Q Xu J Huang J Wang J Zhong W. Targeting of glutamine transporter ASCT2 and glutamine synthetase suppresses gastric cancer cell growth. J Cancer Res Clin Oncol. (2018) 144:821–33. doi: 10.1007/s00432-018-2605-9 PMC5916984 29435734

[87] Matés JM Campos-Sandoval JA Santos-Jiménez JL Márquez J. Dysregulation of glutaminase and glutamine synthetase in cancer. Cancer Lett. (2019) 467:29–39. doi: 10.1016/j.canlet.2019.09.011 31574293

[88] Jing F Hu X Cao Y Xu M Wang Y Jing Y. Discriminating gastric cancer and gastric ulcer using human plasma amino acid metabolic profile. IUBMB Life. (2018) 70:553–62. doi: 10.1002/iub.v70.6 29626382

[89] Ren W Liu G Yin J Tan B Wu G Bazer FW. Amino-acid transporters in T-cell activation and differentiation. Cell Death Dis. (2017) 8:e2655–5. doi: 10.1038/cddis.2016.222 PMC5520724 28471453

[90] Shang Z Ma Z Wu E Chen X Tuo B Li T. Effect of metabolic reprogramming on the immune microenvironment in gastric cancer. Biomed Pharmacother. (2024) 170:116030. doi: 10.1016/j.biopha.2023.116030 38128177

[91] Balaban S Shearer RF Lee LS Geldermalsen van M Schreuder M Shtein HC. Adipocyte lipolysis links obesity to breast cancer growth: adipocyte-derived fatty acids drive breast cancer cell proliferation and migration. Cancer Metab. (2017) 5:1. doi: 10.1186/s40170-016-0163-7 28101337 PMC5237166

[92] Cerne D Melkic E Trost Z Sok M Marc J. Lipoprotein lipase activity and gene expression in lung cancer and in adjacent noncancer lung tissue. Exp Lung Res. (2007) 33:217–25. doi: 10.1080/01902140701481054 17620184

[93] Cao D Song X Che L Li X Pilo MG Vidili G. Both *de novo* synthetized and exogenous fatty acids support the growth of hepatocellular carcinoma cells. Liver Int. (2017) 37:80–9. doi: 10.1111/liv.2017.37.issue-1 PMC5140766 27264722

[94] Dong W Gong H Zhang G Vuletic S Albers J Zhang J. Lipoprotein lipase and phospholipid transfer protein overexpression in human glioma cells and their effect on cell growth, apoptosis, and migration. Acta Biochim Biophys Sin (Shanghai). (2017) 49:62–73. doi: 10.1093/abbs/gmw117 27864281

[95] Cui MY Yi X Zhu DX Wu J. The role of lipid metabolism in gastric cancer. Front Oncol. (2022) 12:916661. doi: 10.3389/fonc.2022.916661 35785165 PMC9240397

[96] Wang C Yang Z Xu E Shen X Wang X Li Z. Apolipoprotein C-II induces EMT to promote gastric cancer peritoneal metastasis via PI3K/AKT/mTOR pathway. Clin Transl Med. (2021) 11:e522. doi: 10.1002/ctm2.v11.8 34459127 PMC8351524

[97] Jiang M Wu N Xu B Chu Y Li X Su S. Fatty acid-induced CD36 expression via O-GlcNAcylation drives gastric cancer metastasis. Theranostics. (2019) 9:5359–73. doi: 10.7150/thno.34024 PMC6691574 31410220

[98] Pan J Fan Z Wang Z Dai Q Xiang Z Yuan F. CD36 mediates palmitate acid-induced metastasis of gastric cancer via AKT/GSK-3β/β-catenin pathway. J Exp Clin Cancer Res. (2019) 38:52. doi: 10.1186/s13046-019-1049-7 30717785 PMC6360779

[99] Lin R Zhang H Yuan Y He Q Zhou J Li S. Fatty acid oxidation controls CD8(+) tissue-resident memory T-cell survival in gastric adenocarcinoma. Cancer Immunol Res. (2020) 8:479–92. doi: 10.1158/2326-6066.CIR-19-0702 32075801

[100] Kumagai S Togashi Y Sakai C Kawazoe A Kawazu M Ueno T. An oncogenic alteration creates a microenvironment that promotes tumor progression by conferring a metabolic advantage to regulatory T cells. Immunity. (2020) 53:187–203.e8. doi: 10.1016/j.immuni.2020.06.016 32640259

[101] Sun L Wang Q Chen B Zhao Y Shen B Wang X. Human gastric cancer mesenchymal stem cell-derived IL15 contributes to tumor cell epithelial-mesenchymal transition via upregulation tregs ratio and PD-1 expression in CD4(+)T cell. Stem Cells Dev. (2018) 27:1203–14. doi: 10.1089/scd.2018.0043 29901436

[102] Shen Y Teng Y Lv Y Zhao Y Qiu Y Chen W. PD-1 does not mark tumor-infiltrating CD8+ T cell dysfunction in human gastric cancer. J Immunother Cancer. (2020) 8. doi: 10.1136/jitc-2019-000422 PMC7406116 32753468

[103] Peng LS Zhang JY Teng YS Zhao YL Wang TT Mao FY. Tumor-associated monocytes/macrophages impair NK-cell function via TGFβ1 in human gastric cancer. Cancer Immunol Res. (2017) 5:248–56. doi: 10.1158/2326-6066.CIR-16-0152 28148545

[104] Chen Y Zhang S Wang Q Zhang X. Tumor-recruited M2 macrophages promote gastric and breast cancer metastasis via M2 macrophage-secreted CHI3L1 protein. J Hematol Oncol. (2017) 10:36. doi: 10.1186/s13045-017-0408-0 28143526 PMC5286803

[105] Lin C He H Liu H Li R Chen Y Qi Y. Tumour-associated macrophages-derived CXCL8 determines immune evasion through autonomous PD-L1 expression in gastric cancer. Gut. (2019) 68:1764–73. doi: 10.1136/gutjnl-2018-316324 30661053

[106] Ju X Zhang H Zhou Z Chen M Wang Q. Tumor-associated macrophages induce PD-L1 expression in gastric cancer cells through IL-6 and TNF-α signaling. Exp Cell Res. (2020) 396:112315. doi: 10.1016/j.yexcr.2020.112315 33031808

[107] Zhu Q Zhang X Zhang L Li W Wu H Yuan X. The IL-6-STAT3 axis mediates a reciprocal crosstalk between cancer-derived mesenchymal stem cells and neutrophils to synergistically prompt gastric cancer progression. Cell Death Dis. (2014) 5:e1295. doi: 10.1038/cddis.2014.263 24946088 PMC4611735

[108] Yang B Su K Sha G Bai Q Sun G Chen H. LINC00665 interacts with BACH1 to activate Wnt1 and mediates the M2 polarization of tumor-associated macrophages in GC. Mol Immunol. (2022) 146:1–8. doi: 10.1016/j.molimm.2022.03.120 35395473

[109] Xin L Wu Y Liu C Zeng F Wang JL Wu DZ. Exosome-mediated transfer of lncRNA HCG18 promotes M2 macrophage polarization in gastric cancer. Mol Immunol. (2021) 140:196–205. doi: 10.1016/j.molimm.2021.10.011 34735868

[110] Xie C Guo Y Lou S. LncRNA ANCR promotes invasion and migration of gastric cancer by regulating foxO1 expression to inhibit macrophage M1 polarization. Dig Dis Sci. (2020) 65:2863–72. doi: 10.1007/s10620-019-06019-1 31894487

[111] Ou J Lei P Yang Z Yang M Luo L Mo H. LINC00152 mediates CD8(+) T-cell infiltration in gastric cancer through binding to EZH2 and regulating the CXCL9, 10/CXCR3 axis. J Mol Histol. (2021) 52:611–20. doi: 10.1007/s10735-021-09967-z 33709190

[112] Miliotis C Slack FJ. miR-105-5p regulates PD-L1 expression and tumor immunogenicity in gastric cancer. Cancer Lett. (2021) 518:115–26. doi: 10.1016/j.canlet.2021.05.037 PMC8355212 34098061

[113] Fang W Shi C Wang Y Song J Zhang L. microRNA-128-3p inhibits CD4+ regulatory T cells enrichment by targeting interleukin 16 in gastric cancer. Bioengineered. (2022) 13:1025–38. doi: 10.1080/21655979.2021.2017566 PMC8805824 34968167

[114] Liang Y Liu Y Zhang Q Zhang H Du J. Tumor-derived extracellular vesicles containing microRNA-1290 promote immune escape of cancer cells through the Grhl2/ZEB1/PD-L1 axis in gastric cancer. Transl Res. (2021) 231:102–12. doi: 10.1016/j.trsl.2020.12.003 33321257

[115] Zhang Y Yang Y Chen Y Lin W Chen X Liu J. PD-L1: Biological mechanism, function, and immunotherapy in gastric cancer. Front Immunol. (2022) 13:1060497. doi: 10.3389/fimmu.2022.1060497 36505487 PMC9729722

[116] Gu L Chen M Guo D Zhu H Zhang W Pan J. PD-L1 and gastric cancer prognosis: A systematic review and meta-analysis. PloS One. (2017) 12:e0182692. doi: 10.1371/journal.pone.0182692 28796808 PMC5552131

[117] Kang YK Boku N Satoh T Ryu MH Chao Y Kato K. Nivolumab in patients with advanced gastric or gastro-oesophageal junction cancer refractory to, or intolerant of, at least two previous chemotherapy regimens (ONO-4538-12, ATTRACTION-2): a randomised, double-blind, placebo-controlled, phase 3 trial. Lancet. (2017) 390:2461–71. doi: 10.1016/S0140-6736(17)31827-5 28993052

[118] Tang Z Wang Y Liu D Wang X Xu C Yu Y. The Neo-PLANET phase II trial of neoadjuvant camrelizumab plus concurrent chemoradiotherapy in locally advanced adenocarcinoma of stomach or gastroesophageal junction. Nat Commun. (2022) 13:6807. doi: 10.1038/s41467-022-34403-5 36357415 PMC9649722

[119] Marabelle A Le DT Ascierto PA Di Giacomo AM De Jesus-Acosta A Delord JP. Efficacy of pembrolizumab in patients with noncolorectal high microsatellite instability/mismatch repair-deficient cancer: results from the phase II KEYNOTE-158 study. J Clin Oncol. (2020) 38:1–10. doi: 10.1200/JCO.19.02105 31682550 PMC8184060

[120] Bang YJ Ruiz EY Van Cutsem E Lee KW Wyrwicz L Schenker M. Phase III, randomised trial of avelumab versus physician’s choice of chemotherapy as third-line treatment of patients with advanced gastric or gastro-oesophageal junction cancer: primary analysis of JAVELIN Gastric 300. Ann Oncol. (2018) 29:2052–60. doi: 10.1093/annonc/mdy264 PMC6225815 30052729

[121] Kang Y-K Terashima M Kim Y.-W Boku N Chung HC Chen J.-S. Adjuvant nivolumab plus chemotherapy versus placebo plus chemotherapy for stage III gastric or gastro-oesophageal junction cancer after gastrectomy with D2 or more extensive lymph-node dissection (ATTRACTION-5): a randomised, multicentre, double-blind, placebo-controlled, phase 3 trial. Lancet Gastroenterol Hepatol. (2024) 9:705–17. doi: 10.1016/S2468-1253(24)00156-0 38906161

[122] Liu T Bai Y Lin X Li W Wang J Zhang X. First-line nivolumab plus chemotherapy vs chemotherapy in patients with advanced gastric, gastroesophageal junction and esophageal adenocarcinoma: CheckMate 649 Chinese subgroup analysis. Int J Cancer. (2023) 152:749–60. doi: 10.1002/ijc.v152.4 PMC10092493 36121651

[123] Dayyani F Chao J Lee FC Taylor TH Neumann K Cho MT. A phase II study of cabozantinib and pembrolizumab in advanced gastric/gastroesophageal adenocarcinomas resistant or refractory to immune checkpoint inhibitors. Oncologist. (2024) 29:721–e1088. doi: 10.1093/oncolo/oyae117 38823034 PMC11299925

[124] Romo-Tena J Gómez-Martín D Alcocer-Varela J. CTLA-4 and autoimmunity: new insights into the dual regulator of tolerance. Autoimmun Rev. (2013) 12:1171–6. doi: 10.1016/j.autrev.2013.07.002 23851140

[125] Kim GR Choi JM. Current understanding of cytotoxic T lymphocyte antigen-4 (CTLA-4) signaling in T-cell biology and disease therapy. Mol Cells. (2022) 45:513–21. doi: 10.14348/molcells.2022.2056 PMC9385567 35950451

[126] Bang YJ Cho JY Kim YH Kim JW Di Bartolomeo M Ajani JA. Efficacy of sequential ipilimumab monotherapy versus best supportive care for unresectable locally advanced/metastatic gastric or gastroesophageal junction cancer. Clin Cancer Res. (2017) 23:5671–8. doi: 10.1158/1078-0432.CCR-17-0025 28655793

[127] Kelly RJ Lee J Bang YJ Almhanna K Blum-Murphy M Catenacci DVT. Safety and efficacy of durvalumab and tremelimumab alone or in combination in patients with advanced gastric and gastroesophageal junction adenocarcinoma. Clin Cancer Res. (2020) 26:846–54. doi: 10.1158/1078-0432.CCR-19-2443 PMC7748730 31676670

[128] Hu BS Tang T Jia JL Xie BC Wu TL Sheng YY. CD137 agonist induces gastric cancer cell apoptosis by enhancing the functions of CD8(+) T cells via NF-κB signaling. Cancer Cell Int. (2020) 20:513. doi: 10.1186/s12935-020-01605-0 33093811 PMC7576737

[129] Zhu B Sun Y Wei X Zhou H Cao J Li C. Dendritic cell vaccine loaded with MG-7 antigen induces cytotoxic T lymphocyte responses against gastric cancer. J Healthc Eng. (2022) 2022:1964081. doi: 10.1155/2022/1964081 35480145 PMC9038393

[130] Zhou Z Tao C Li J Tang JC Chan AS Zhou Y. Chimeric antigen receptor T cells applied to solid tumors. Front Immunol. (2022) 13:984864. doi: 10.3389/fimmu.2022.984864 36389701 PMC9659902

[131] Johnson LA June CH. Driving gene-engineered T cell immunotherapy of cancer. Cell Res. (2017) 27:38–58. doi: 10.1038/cr.2016.154 28025979 PMC5223234

[132] Bębnowska D Grywalska E Niedźwiedzka-Rystwej P Sosnowska-Pasiarska B Smok-Kalwat J Pasiarski M. CAR-T cell therapy-an overview of targets in gastric cancer. J Clin Med. (2020) 9:1894. doi: 10.3390/jcm9061894 32560392 PMC7355670

[133] Song Y Tong C Wang Y Gao Y Dai H Guo Y. Effective and persistent antitumor activity of HER2-directed CAR-T cells against gastric cancer cells *in vitro* and xenotransplanted tumors *in vivo* . Protein Cell. (2018) 9:867–78. doi: 10.1007/s13238-017-0384-8 PMC6160382 28284008

[134] Jung M Yang Y McCloskey JE Zaman M Vedvyas Y Zhang X. Chimeric antigen receptor T cell therapy targeting ICAM-1 in gastric cancer. Mol Ther Oncolytics. (2020) 18:587–601. doi: 10.1016/j.omto.2020.08.009 32995483 PMC7501410

[135] Feng Z He X Zhang X Wu Y Xing B Knowles A. Author Correction: Potent suppression of neuroendocrine tumors and gastrointestinal cancers by CDH17CAR T cells without toxicity to normal tissues. Nat Cancer. (2024) 5:691. doi: 10.1038/s43018-024-00766-5 38605236

[136] Lv J Zhao R Wu D Zheng D Wu Z Shi J. Mesothelin is a target of chimeric antigen receptor T cells for treating gastric cancer. J Hematol Oncol. (2019) 12:18. doi: 10.1186/s13045-019-0704-y 30777106 PMC6380000

[137] Lyons TG Ku GY. Systemic therapy for esophagogastric cancer: targeted therapies. Chin Clin Oncol. (2017) 6:48. doi: 10.21037/cco.2017.07.02 29129088

[138] Jiang H Shi Z Wang P Wang C Yang L Du G. Claudin18.2-specific chimeric antigen receptor engineered T cells for the treatment of gastric cancer. J Natl Cancer Inst. (2019) 111:409–18. doi: 10.1093/jnci/djy134 30203099

[139] Johnson LA Morgan RA Dudley ME Cassard L Yang JC Hughes MS. Gene therapy with human and mouse T-cell receptors mediates cancer regression and targets normal tissues expressing cognate antigen. Blood. (2009) 114:535–46. doi: 10.1182/blood-2009-03-211714 PMC2929689 19451549

[140] Looi CK Chung FF Leong CO Wong SF Rosli R Mai CW. Therapeutic challenges and current immunomodulatory strategies in targeting the immunosuppressive pancreatic tumor microenvironment. J Exp Clin Cancer Res. (2019) 38:162. doi: 10.1186/s13046-019-1153-8 30987642 PMC6463646

[141] Lee DW Santomasso BD Locke FL Ghobadi A Turtle CJ Brudno JN. ASTCT consensus grading for cytokine release syndrome and neurologic toxicity associated with immune effector cells. Biol Blood Marrow Transplant. (2019) 25:625–38. doi: 10.1016/j.bbmt.2018.12.758 PMC12180426 30592986

[142] Kloss CC Condomines M Cartellieri M Bachmann M Sadelain M. Combinatorial antigen recognition with balanced signaling promotes selective tumor eradication by engineered T cells. Nat Biotechnol. (2013) 31:71–5. doi: 10.1038/nbt.2459 PMC5505184 23242161

[143] Bielamowicz K Fousek K Byrd TT Samaha H Mukherjee M Aware N. Trivalent CAR T cells overcome interpatient antigenic variability in glioblastoma. Neuro Oncol. (2018) 20:506–18. doi: 10.1093/neuonc/nox182 PMC5909636 29016929

[144] Qin H Ramakrishna S Nguyen S Fountaine TJ Ponduri A Stetler-Stevenson M. Preclinical development of bivalent chimeric antigen receptors targeting both CD19 and CD22. Mol Ther Oncolytics. (2018) 11:127–37. doi: 10.1016/j.omto.2018.10.006 PMC6300726 30581986

[145] Kloss CC Lee J Zhang A Chen F Melenhorst JJ Lacey SF. Dominant-negative TGF-β Receptor enhances PSMA-targeted human CAR T cell proliferation and augments prostate cancer eradication. Mol Ther. (2018) 26:1855–66. doi: 10.1016/j.ymthe.2018.05.003 PMC6037129 29807781

[146] Jung BK Ko HY Kang H Hong J Ahn HM Na Y. Relaxin-expressing oncolytic adenovirus induces remodeling of physical and immunological aspects of cold tumor to potentiate PD-1 blockade. J Immunother Cancer. (2020) 8. doi: 10.1136/jitc-2020-000763 PMC7406118 32753544

[147] Sugawara K Iwai M Yajima S Tanaka M Yanagihara K Seto Y. Efficacy of a third-generation oncolytic herpes virus G47Δ in advanced stage models of human gastric cancer. Mol Ther Oncolytics. (2020) 17:205–15. doi: 10.1016/j.omto.2020.03.022 PMC7178322 32346610

[148] Yang Y Yang Y Chen M Chen J Wang J Ma Y. Injectable shear-thinning polylysine hydrogels for localized immunotherapy of gastric cancer through repolarization of tumor-associated macrophages. Biomater Sci. (2021) 9:6597–608. doi: 10.1039/D1BM01053K 34582523

[149] Liu Q Zhang D Qian H Chu Y Yang Y Shao J. Superior antitumor efficacy of IFN-α2b-incorporated photo-cross-linked hydrogels combined with T cell transfer and low-dose irradiation against gastric cancer. Int J Nanomed. (2020) 15:3669–80. doi: 10.2147/IJN.S249174 PMC7261665 32547021

[150] Xue G Cheng Y Ran F Li X Huang T Yang Y. SLC gene-modified dendritic cells mediate T cell-dependent anti-gastric cancer immune responses *in vitro* . Oncol Rep. (2013) 29:595–604. doi: 10.3892/or.2012.2154 23229068

[151] Chen Y Chen B Yang T Xiao W Qian L Ding Y. Human fused NKG2D-IL-15 protein controls xenografted human gastric cancer through the recruitment and activation of NK cells. Cell Mol Immunol. (2017) 14:293–307. doi: 10.1038/cmi.2015.81 26364916 PMC5360879

[152] Ponziani S Di Vittorio G Pitari G Cimini AM Ardini M Gentile R. Antibody-drug conjugates: the new frontier of chemotherapy. Int J Mol Sci. (2020) 21:5510. doi: 10.3390/ijms21155510 32752132 PMC7432430

[153] Shitara K Iwata H Takahashi S Tamura K Park H Modi S. Trastuzumab deruxtecan (DS-8201a) in patients with advanced HER2-positive gastric cancer: a dose-expansion, phase 1 study. Lancet Oncol. (2019) 20:827–36. doi: 10.1016/S1470-2045(19)30088-9 31047804

[154] Ricci AD Rizzo A Llimpe Rojas FL Di Fabio F De Biase D Rihawi K. Novel HER2-directed treatments in advanced gastric carcinoma: anotHER paradigm shift? Cancers (Basel). (2021) 13:1664. doi: 10.3390/cancers13071664 33916206 PMC8036476

[155] Xu Y Wang Y Gong J Zhang X Peng Z Sheng X. Phase I study of the recombinant humanized anti-HER2 monoclonal antibody-MMAE conjugate RC48-ADC in patients with HER2-positive advanced solid tumors. Gastric Cancer. (2021) 24:913–25. doi: 10.1007/s10120-021-01168-7 PMC8205919 33945049

[156] Peng Z Liu T Wei J Wang A He Y Yang L. Efficacy and safety of a novel anti-HER2 therapeutic antibody RC48 in patients with HER2-overexpressing, locally advanced or metastatic gastric or gastroesophageal junction cancer: a single-arm phase II study. Cancer Commun (Lond). (2021) 41:1173–82. doi: 10.1002/cac2.v41.11 PMC8626607 34665942

[157] Wang Y Gong J Wang A Wei J Peng Z Wang X. Disitamab vedotin (RC48) plus toripalimab for HER2-expressing advanced gastric or gastroesophageal junction and other solid tumours: a multicentre, open label, dose escalation and expansion phase 1 trial. EClinicalMedicine. (2024) 68:102415. doi: 10.1016/j.eclinm.2023.102415 38235421 PMC10789637

[158] Nakamura Y Kinoshita J Yamaguchi T Aoki T Saito H Hamabe-Horiike T. Crosstalk between cancer-associated fibroblasts and immune cells in peritoneal metastasis: inhibition in the migration of M2 macrophages and mast cells by Tranilast. Gastric Cancer. (2022) 25:515–26. doi: 10.1007/s10120-021-01275-5 PMC9013333 34997450

[159] Sootome H Fujita H Ito K Ochiiwa H Fujioka Y Ito K. Futibatinib is a novel irreversible FGFR 1-4 inhibitor that shows selective antitumor activity against FGFR-deregulated tumors. Cancer Res. (2020) 80:4986–97. doi: 10.1158/0008-5472.CAN-19-2568 32973082

[160] Chen G Yu C Tang Z Liu S An F Zhu J. Metformin suppresses gastric cancer progression through calmodulin−like protein 3 secreted from tumor−associated fibroblasts. Oncol Rep. (2019) 41:405–14. doi: 10.3892/or.2018.6783 30320344

[161] Luo Q Zheng N Jiang L Wang T Zhang P Liu Y. Lipid accumulation in macrophages confers protumorigenic polarization and immunity in gastric cancer. Cancer Sci. (2020) 111:4000–11. doi: 10.1111/cas.v111.11 PMC7648032 32798273

[162] Wang X Jiao X Meng Y Chen H Griffin N Gao X. Methionine enkephalin (MENK) inhibits human gastric cancer through regulating tumor associated macrophages (TAMs) and PI3K/AKT/mTOR signaling pathway inside cancer cells. Int Immunopharmacol. (2018) 65:312–22. doi: 10.1016/j.intimp.2018.10.023 30343258

